# Optimizing Aggregated N-Of-1 Trial Designs for Predictive Biomarker Validation: Statistical Methods and Theoretical Findings

**DOI:** 10.3389/fdgth.2020.00013

**Published:** 2020-08-28

**Authors:** Rebecca C. Hendrickson, Ronald G. Thomas, Nicholas J. Schork, Murray A. Raskind

**Affiliations:** ^1^VISN 20 Northwest Network Mental Illness Research, Education and Clinical Center (MIRECC), VA Puget Sound Health Care System, Seattle, WA, United States; ^2^Department of Psychiatry and Behavioral Sciences, University of Washington School of Medicine, Seattle, WA, United States; ^3^Department of Biostatistics, University of California, San Diego, San Diego, CA, United States; ^4^Quantitative Medicine and Systems Biology, The Translational Genomics Research Institute (TGen), Phoenix, AZ, United States; ^5^The Joint City of Hope/TGen IMPACT Center (NJS), City of Hope National Medical Center, Duarte, CA, United States

**Keywords:** N-of-1 trials, crossover trials, posttraumatic stress disorder (PTSD), prazosin, biomarkers, personalized medicine

## Abstract

**Background and Significance:** Parallel-group randomized controlled trials (PG-RCTs) are the gold standard for detecting differences in mean improvement across treatment conditions. However, PG-RCTs provide limited information about individuals, making them poorly optimized for quantifying the relationship of a biomarker measured at baseline with treatment response. In N-of-1 trials, an individual subject moves between treatment conditions to determine their specific response to each treatment. Aggregated N-of-1 trials analyze a cohort of such participants, and can be designed to optimize both statistical power and clinical or logistical constraints, such as allowing all participants to begin with an open-label stabilization phase to facilitate the enrollment of more acutely symptomatic participants. Here, we describe a set of statistical simulation studies comparing the power of four different trial designs to detect a relationship between a predictive biomarker measured at baseline and subjects' specific response to the PTSD pharmacotherapeutic agent prazosin.

**Methods:** Data was simulated from 4 trial designs: (1) open-label; (2) open-label + blinded discontinuation; (3) traditional crossover; and (4) open label + blinded discontinuation + brief crossover (the N-of-1 design). Designs were matched in length and assessments. The primary outcome, analyzed with a linear mixed effects model, was whether a statistically significant association between biomarker value and response to prazosin was detected with 5% Type I error. Simulations were repeated 1,000 times to determine power and bias, with varied parameters.

**Results:** Trial designs 2 & 4 had substantially higher power with fewer subjects than open label design. Trial design 4 also had higher power than trial design 2. Trial design 4 had slightly lower power than the traditional crossover design, although power declined much more rapidly as carryover was introduced.

**Conclusions:** These results suggest that an aggregated N-of-1 trial design beginning with an open label titration phase may provide superior power over open label or open label and blinded discontinuation designs, and similar power to a traditional crossover design, in detecting an association between a predictive biomarker and the clinical response to the PTSD pharmacotherapeutic prazosin. This is achieved while allowing all participants to spend the first 8 weeks of the trial on open-label active treatment.

## Introduction

Parallel-group randomized controlled trials (PG-RCTs) are the gold standard for detecting differences in mean improvement across treatment conditions (1). However, PG-RCTs provide limited information about the response of individuals to treatment, as they provide no information about the potential response to active treatment for those in the placebo group, and for those who do receive active treatment and experience clinical improvement, it is not possible to distinguish whether this improvement is treatment-specific, or whether the individual would have responded similarly to placebo. This makes PG-RCTs poorly optimized for quantifying the relationship of a biomarker measured at baseline to a treatment-specific response, or identifying subgroups of treatment-specific response (2).

These limitations also affect the utility of trial participation for participants, who receive limited information about whether they have a treatment-specific response (1, 3). Additionally, this trial design requires that many participants spend the full duration of the study on placebo, which may limit the enrollment of patients with particularly acute symptoms. The risk of under enrolling acutely symptomatic patients in a PG-RCT may be particularly high in contexts where the treatment in question or treatments very similar to it are already in active clinical use (4), as is often the case in clinical trials designed to address questions in the realm of personalized medicine.

In N-of-1 trials, an individual subject experiences several treatment conditions, such as active treatment and placebo, in order to assess the individual's specific response to each treatment (1). In aggregated N-of-1 trials, a cohort of individuals moves through this same type of trial design, and their outcomes are analyzed to answer questions about e.g., patterns of treatment response (5). Aggregated N-of-1 trials can be designed to optimize both statistical power and clinical or logistical constraints, such as allowing all participants to begin with an open-label stabilization phase to facilitate the enrollment of more acutely symptomatic participants. They can also mix elements that facilitate standardized assessment of change across all participants with evaluative elements that are individualized to address symptoms that are specific or important to individual participants (1, 6). These features suggest that aggregated N-of-1 clinical trial designs may have significant advantages over PG-RCTs in addressing hypotheses related to personalized medicine (2, 7).

Despite these potential advantages, N-of-1 trials have been slow to gain traction in the biomedical research community. One reason may be that N-of-1 trials have statistical complexities that are different from those encountered in PG-RCTs, and their design and utilization has been limited by the availability of statistical methods to validate and interpret the results (1). Not only do standard methods of power calculations not apply to an aggregated N-of-1 clinical trial, but the breadth of trial designs that are possible using an aggregated N-of-1 approach mean that the questions a researcher would like to ask when computing power calculations may differ from those asked when designing PG-RCTs. For example, the power of an aggregated N-of-1 clinical trial generally increases with increasing repetitions of each treatment condition (5). This effect is limited, however, by the fact that the shorter the period of time an individual is on a given treatment before the effect of that treatment is measured and the treatment condition changed, the larger any carry-over effects from the previous treatment blocks are likely to be (5). The relative cost- vs. benefit of longer but fewer total blocks of treatment, vs. shorter but a larger number of blocks of treatment, then, will depend on the researcher's estimate of carry-over in their particular experimental context (8)–and it is important that methods for power calculations for aggregated N-of-1 trial designs take this type of a factor into account.

Another area in which the assessment of power in an aggregated N-of-1 trial may be more complex is in the area of drop-outs. In traditional power calculation methods, it is often hopefully assumed that dropouts will be unbiased with respect to the effects being measured (9); when the risk of biased drop outs is addressed, this is usually done during analysis by using last-measure-carried-forward, multiple-imputation, or similar strategies (10). In a clinical trial design where participants will at some point move from active treatment to placebo, or from one treatment condition to another treatment condition, it becomes harder to ignore the likelihood that those who have the strongest response to one particular treatment condition may be the most likely to drop out when the move from that treatment condition to one that is less effective for them (11). At the same time, the increased flexibility of the trial design means that it is may be possible to explicitly structure a clinical trial to both minimize dropouts and maximize the ability to obtain the most critical information from each participant prior to periods where the likelihood of dropout increases, if these factors can be quantified and compared across potential clinical trial designs.

Finally, while the option to include both open-label and blinded treatment blocks into an aggregated N-of-1 trial design has the potential to significantly increase the representation of acutely symptomatic patients in a clinical trial, it also makes assessing the impact of a participant's expectation of benefit on their outcome more complex than in a purely double-blinded RCT (5). For example, if a participant begins on open-label treatment, it is expected that their change in outcome measurements during this period of time would constitute the combined effect of both treatment-specific effects and non-treatment-specific effects, which includes the impact of just knowing that they are receiving active treatment. The question arises, then, as to what is expected to happen when they transition from this period of open-label treatment to a treatment block when they are on blinded but active treatment. How does the impact of knowing they *may* be on treatment compare to the impact of knowing they *are* on treatment?

The increased relevance of factors such as biased dropouts and expectancy related effects to statistical power means that wider adoption of aggregated N-of-1 clinical trial designs will require the development of statistical methods that allow clinical trialists to compare the statistical power of different potential trial designs in answering their particular research questions, and given their best estimates of the extent to which effects such as carry-over or biased dropout rates will impact their study population. As many of these factors do have non-trivial relevance even to PG-RCTs, however (12, 13), it is also possible that the development and such methods may eventually improve our understanding and interpretation of more traditional clinical trial designs, as well.

Although the simplest form of an N-of-1 trial, the crossover trial, is one of the earliest forms of clinical trial and has been studied extensively (14–17), most work addressing the statistical properties of more complex N-of-1 clinical trial designs has been done in the past decade (5, 8). In 2014, Chen and Chen compared both simple (paired *t*-test) and more complex (mixed effects models) approaches for conducting tests of treatment efficacy using aggregated N-of-1 trial results, and found that in their examples, mixed effects models were inferior in the absence of carryover effects but superior when these were included (18). This work was critiqued by Araujo et al. who point out that the models evaluated by Chen and Chen do not include a treatment by patient interaction (19), an interaction that has been advocated for in the meta-analysis literature; the relevance of Chen and Chen's approach may also be limited by the assumption of compound symmetry and auto-regressive covariance structure. More recently, Percha et al. implemented a stochastic time-series model to simulate individual N-of-1 studies, and characterized the impact of the number of treatment blocks, the ordering of treatments within blocks, the duration of each treatment, and the sampling frequency on both the statistical power to detect a difference in efficacy and in the accuracy of the estimated effect size (20). However, little work thus far has explicitly attempted to model the impact of expectancy and biased dropout on statistical power in aggregated N-of-1 clinical trial design, or to incorporate the possibility of non-traditional combinations of treatment conditions, such as trials that include both open label and blinded conditions, or blinded discontinuation blocks.

Finally, although it is expected to be an important application of this type of trial design (2, 7), there is extremely little that has been published addressing methods for the validation of predictive biomarkers in aggregated N-of-1 trials. A publication by Grenet et al. earlier this year provides a statistical framework for comparing the power of crossover vs. parallel-group clinical trials to detect a relationship between a binary predictive biomarker and treatment effect (21). However, we are not aware of any published methods for analyzing more complex aggregated N-of-1 clinical trials to test for the relationship between a putative predictive biomarker and treatment response, nor for calculating a trial's power to test this type of a hypothesis.

Here, we provide an initial set of tools designed to address a number of the above statistical challenges in the design and analysis of aggregated N-of-1 trials. Specifically, we describe a set of statistical simulation studies that were used to compare the expected statistical power of different potential clinical trial designs, the aim of which was to detect a relationship between a biomarker measured at entry into the study and the efficacy of a specific treatment. Importantly, then, the power that is being calculated in this set of examples does not address whether the treatment is effective as compared with placebo, but rather whether the biomarker measured at baseline is able to predict which individuals will respond to the treatment and which will not.

This sample application is based on work conducted by the authors to plan a randomized clinical trial to test the relationship between standing systolic blood pressure measured at baseline to the decrease in PTSD symptoms produced by the α_1_ adrenoceptor antagonist prazosin. A relationship between this simple, clinically-accessible biomarker and treatment response that is large enough to be potentially relevant to treatment selection has been found in a *post hoc* analysis of a PG-RCT of prazosin for PTSD conducted in a primarily young, male population (22), but the relationship has not yet been validated in a prospective trial, or in a trial with a less homogeneous population. Further, the potential to conduct further PG-RCTs of prazosin for PTSD is believed to be limited by already wide utilization of prazosin for PTSD, such that the acutely symptomatic patients thought to be most responsive to PTSD are unlikely to be referred to trials where they may be placed on placebo, rather than simply treated (4).

The use of this real example of computing power calculations for what is now an ongoing aggregated N-of-1 clinical trial allows us to demonstrate how estimates of population means and variances were extracted from extant data sets when possible, while variables that could not be estimated based on existing data were allowed to vary so that the dependence of the power calculations on these estimates could be assessed. However, it is hoped that the methods described will be of general utility. To this end, the functions used to generate and run these simulations are also provided in a publicly available github repository.

## Methods

### Approach

**Figure 1:**
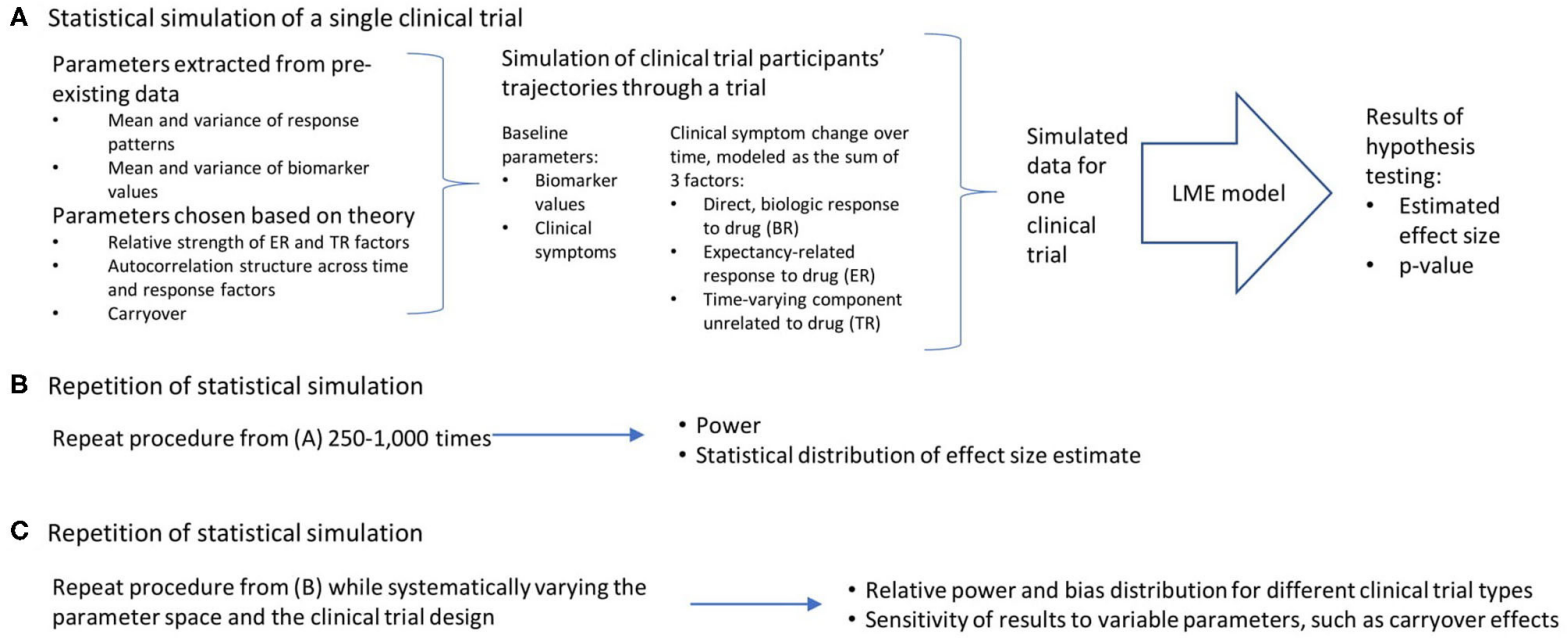
Schematic overview of approach to simulating and analyzing clinical trials data. **(A)** A multi-step process was used to simulate and analyze the results of a single simulated clinical trial. Parameters used to generate the simulated data were derived in part from existing data sets, but also involved the selection of some parameters that could not be estimated directly from existing data. The generation of data was done using a model that presumed there were three basic factors that linearly combine to describe the trajectory of participants' symptoms over time (the direct, biologic response to drug (BR), the expectancy-related response to drug (ER), and the time-varying component unrelated to drug (TR). These results were then analyzed using a linear mixed effects model, as is proposed for the analysis of the actual clinical trial results. This analysis is structured to test the hypothesis that the biomarker measured at baseline will be significantly associated with the degree of clinical response a given participant has to the intervention. The analysis produces a *p*-value describing the statistical significance of the results if they were being analyzed as a single extant clinical trial, and an estimate of effect size. **(B)** This simulation process is repeated 1,000 times using the same clinical trial design and parameter selection, allowing an estimate of the power of this trial design to detect the proposed relationship, and the distribution of bias in the effect size estimated. **(C)** This entire process can then be repeated with (a) different clinical trial designs, and (b) different parameter selection, in order to determine how statistical power and bias in effect size estimation vary as a function of trial design, response parameters, and model assumptions.

Conceptually, the work can be broken down into three broad steps, which are detailed in Figure 1: the statistical simulation of a single clinical trial, including a simulated data set and the estimated effect size and p-value that result from the analysis of that trial (Figure 1A); the repetition of the individual clinical trial simulation 1,000 times, producing an estimate of statistical power and the distribution of bias in the effect size estimate (Figure 1B); and a repetition of this entire process while systematically varying the parameter space and the clinical trial design, in order to quantify the relative power and bias distributions for the different clinical trial designs, and the sensitivity of these results to variable parameters such as carryover effects of dropout patterns (Figure 1C). All work was done using R (23) and RStudio (24). The R functions and vignettes documenting the steps used to generate these results are available as a package at https://github.com/rchendrickson/pmsimstats.

### Selection of Clinical Trial Designs for Comparison

**Figure 2:**
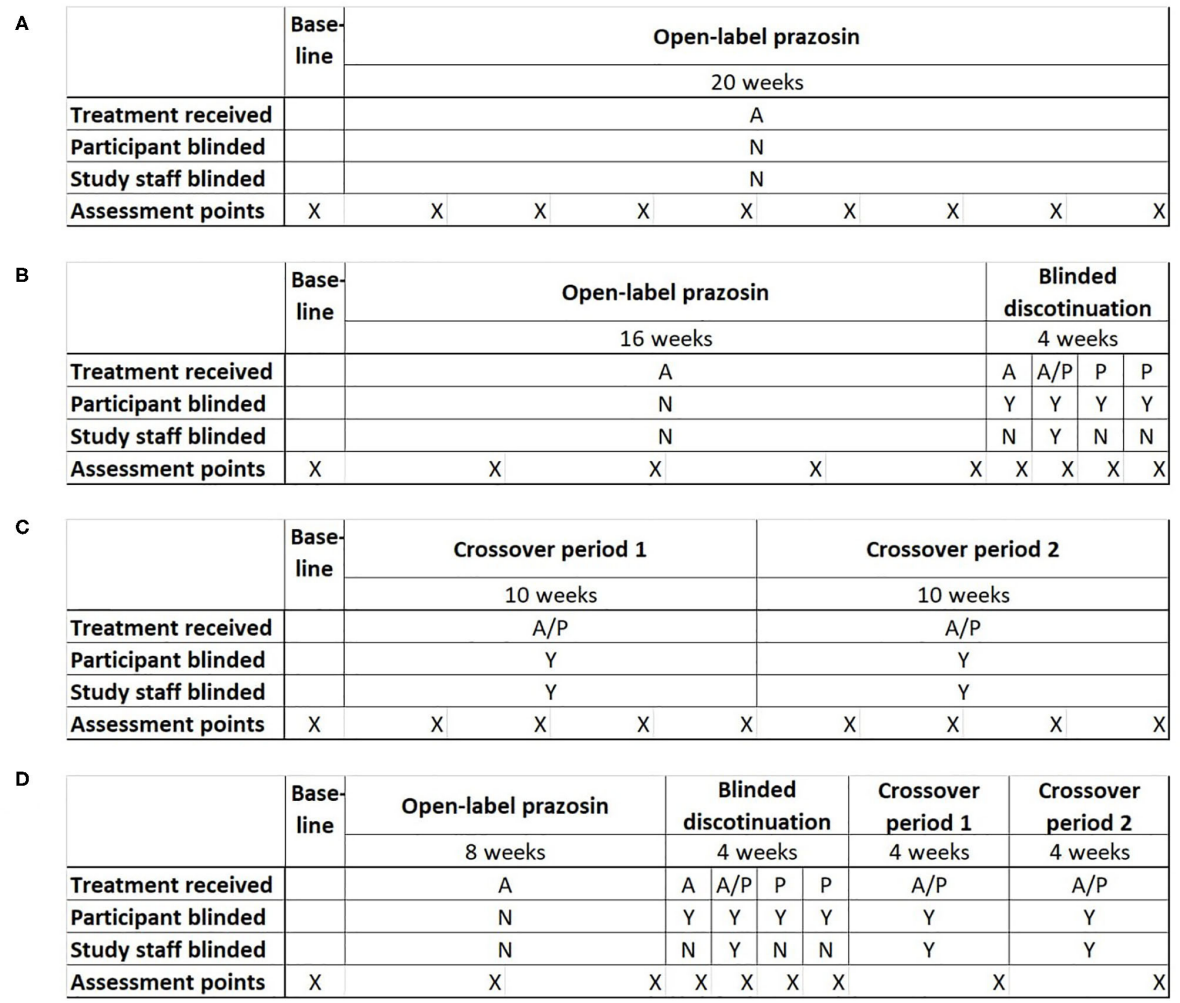
Four potential clinical trial designs that were compared on their ability to detect a relationship between a biomarker measured at baseline and response to treatment with the pharmacotherapeutic agent prazosin. Trial designs were matched in duration and the number of evaluation points. **(A)** Open-label trial design: All participants receive open-label prazosin throughout the trial. **(B)** Open-label followed by blinded discontinuation design: All participants receive active drug for 16 weeks, then enter a 4 week blinded discontinuation block. During the blinded discontinuation block, all participants receive active drug during the first week and placebo during the last 2 weeks, such that only the participant is blinded to treatment condition during these weeks; the treatment condition during the second week is randomly assigned and a double blind is maintained during this week. **(C)** Traditional crossover trial design: Participants are randomized to 10 weeks on active drug followed by 10 weeks on placebo, or the reverse. **(D)** Proposed N-of-1 trial design: all participants begin with an 8 week open label period, then enter a 4 week blinded discontinuation period, then complete 8 weeks of crossover. There are two independent randomization points—whether the participant is on active drug (A) or placebo (P) during the second week of the blinded discontinuation block, and whether the participant's crossover blocks are active drug then placebo or the reverse. A = Active drug (prazosin); P = placebo. X indications the timing of assessment points for clinical outcome measure.

Potential clinical trial designs were selected to allow the comparison of statistical power and bias across the four most plausible trial designs for testing the relationship of a baseline biomarker to treatment response: (1) a single-group open label trial, (2) a single-group open label trial followed by a blinded discontinuation block, (3) a traditional crossover trial, and (4) the proposed N-of-1 trial design, consisting of a single-group open label trial followed by first a blinded discontinuation block and then two crossover blocks (Figure 2). In each design, the titration period for prazosin is expected to be 2.5 weeks each time it is initiated. In blinded discontinuation blocks, the transition from blinded but active treatment to placebo can occur at only two points, either after 1 week or after 2 weeks in the block. However, this aspect of the design is not revealed to participants, who are told only that during this block they may be on either active drug or placebo, and that this may change during the course of the block.

The inclusion of an open-label period at the beginning of designs 2 and 4 was selected to address concerns that highly symptomatic individuals would be less likely to be referred to or enroll in a clinical trial where they may be initially assigned to a placebo group. The inclusion of a blinded discontinuation period in two of the trial designs was designed to allow a higher intensity of data capture, including of personalized assessment measures, during the period of discontinuation after the open-label portion. A traditional PG-RCT was not included among the tested designs because, in the specific example being explored, existing data had already demonstrated that there was a negligible chance that the biomarker predicted response to placebo (22), which meant that minimal information would be obtained from the ~50% of participants randomized to placebo.

By the most general definition of an aggregated N-of-1 clinical trial, all but the open label and PG-RCT designs can be considered to be a form of N-of-1 trial, because each participant spends time on both treatment conditions (active drug and control). However, it is primarily the fourth trial design that takes advantage of the opportunity for multiple periods of treatment in each treatment condition.

### Statistical Simulation of Data

The expected trajectory of clinical symptoms over time was modeled as the linear sum of 3 factors (Figure 1A), each of which describes one aspect of how symptoms change over time from their baseline values: (1) a direct, biologic response to a pharmacologic agent (the biologic response, or BR); (2) an expectancy-related response to taking a medication that is either known to be or know to possibly be an active treatment (the expectancy-related response, or ER); and (3) a component that is a function of time since study entry, but which is not related to the actual or expected presence of active treatment (the time-dependent response, or TR). The time-dependent response is presumed to include both regression-to-the-mean effects and the impact of the structure, attention and regular interaction with staff involved in study participation.

A function describing the expected mean and variance of each factor as a function of time and study design was fit using a three-parameter gompertz function, allowing a non-linear monotonic trajectory over time with a maximum asymptote. The three parameters characterize: the maximum response, the displacement, and the rate. Initial estimates for these variables were based on fits to existing data from a parallel group randomized controlled trial of prazosin for PTSD in active duty service members (25), utilizing the following assumptions: the trajectory of the BR factor was taken to be the difference between the trajectory of the prazosin group and the placebo group; the trajectory of the placebo group was taken to represent the sum of the TR and ER factors; in the absence of any data to separate the trajectory of the placebo group into the TR and ER components, the maximum response, rate and variance of the TR and ER factors were assumed to be equal [tabula rasa (TaRa) parameter set]. In further sensitivity analyses, however, these values were varied to assess the impact of these parameters on simulated clinical trial performance. The means and variances of the baseline symptom intensity [as measured by the clinician administered PTSD scale for DSM-IV, or CAPS-IV (26)] and baseline biomarker values (systolic blood pressure 2 min after standing) were based directly on the baseline measurements from the existing data.

The ER factor was presumed to be scaled directly by participant expectancy regarding whether they were taking an active medication or not. For open label trial components, the expectancy was set to 1, while for blinded portions where the participant had been informed there was an equal chance they were taking active drug vs. placebo the expectancy was set to 0.5. The BR factor was set to zero at times when participants had never been on active drug; however, a carryover effect was built in such that when a participant moved from active drug to being off active drug, the value of the BR at the last timepoint on active drug was exponentially decayed, with the half-life of this decay being maintained as a model parameter.

**Figure 3:**
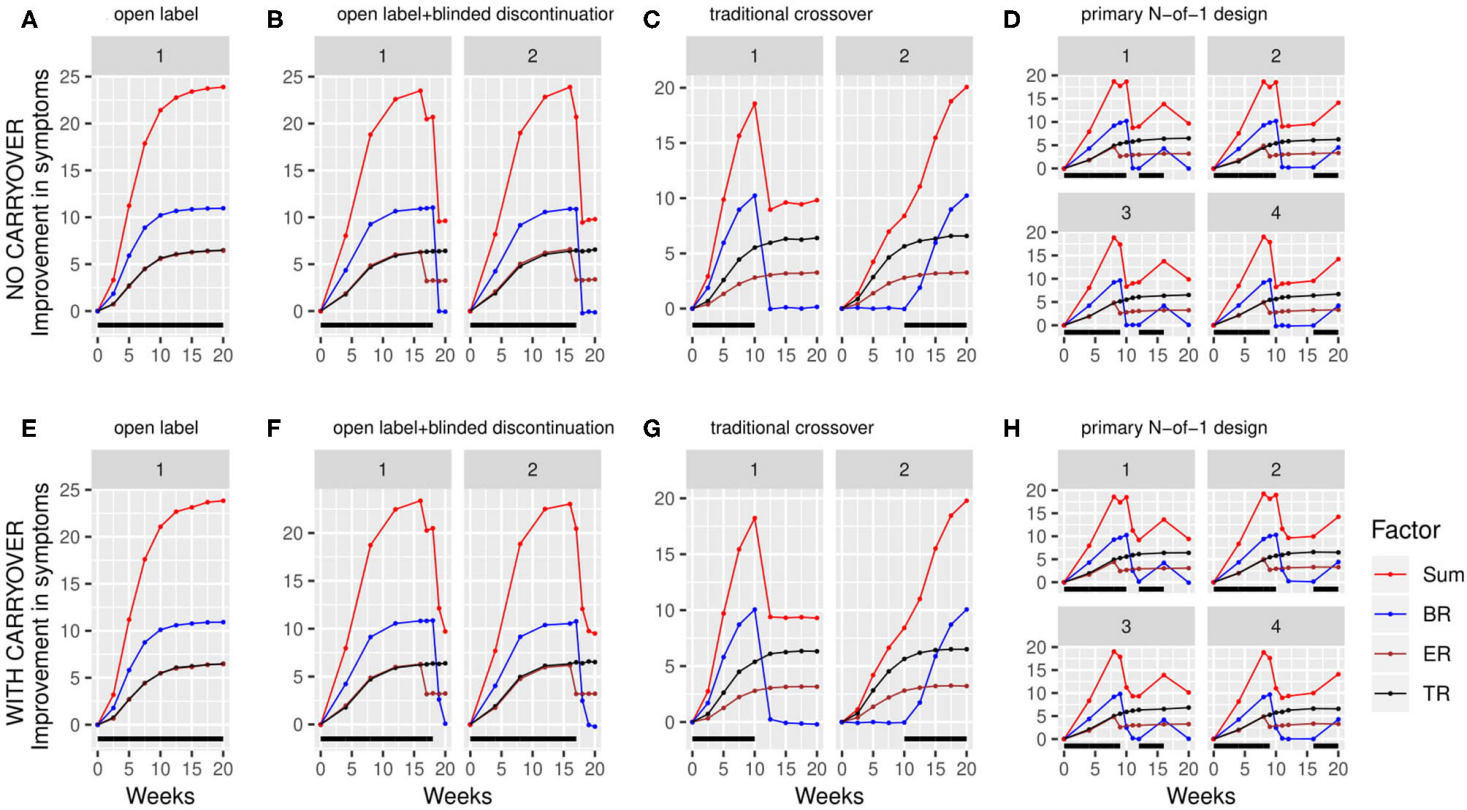
Simulated clinical trials data plotted as change in baseline symptom score as a function of time, broken down by clinical trial design **(A–H)** and randomization path (numbered facets within each trial design). **(A)** through **(D)** show results with carryover set to 0; **(E)** through **(H)** show results with carryover set to 0.1 weeks. Individual simulated factors (BR = biologic response to drug, ER = expectancy-related response to drug, and TR = time-varying component unrelated to drug) are shown along with their summed effect. Plotted data represents the averaged output of 500 replicates for each clinical trial design, divided across the number of randomization paths, and was generated using the tabula rasa response parameters. OL = open label, OL+BDC = open label followed by blinded discontinuation, CO = cross over, N-of-1 = proposed N-of-1 trial design. Black bar represents times active drug was scheduled to be present.

Using the above factor parameterizations to provide the expected mean and variance for each factor at each time point, simulated data with the specified covariance structure, coerced to be positive definite, was generated using the function *mvrnorm* from the R package MASS (27). This simulated data consisted of baseline symptom intensity, baseline biomarker value, and the value of each of the three factors at each timepoint within the trial for a variable number (N) of participants (Figure 3). The *mvrnorm* function takes as input a vector specifying the means of each variable, as well as a covariance matrix. The covariance matrix was assembled based on a set of modifiable parameters defining the correlation between the baseline biomarker and the BR components, the autocorrelation over time (relating the value of a factor for one participant at one timepoint to the value of that factor for that participant at subsequent timepoints), the correlation at a single time point between the 3 factors, and the variance of each component. Once the factor values at each time point were generated, the sums of the three factors BR, ER and TR were subtracted from the baseline values for each simulated participant to produce a full set of results for the simulated clinical trial, consisting of the baseline biomarker measurement and symptom intensity measurement at each timepoint.

In some simulations, a censoring filter was applied following the production of the stimulated trajectories, in order to assess the effects of participant dropout. The probability of a simulated participant dropping out per unit time was calculated as the sum of a flat hazard function (β_0_,) and a probability scaled by the square of the change in symptoms since baseline (shifted by 100 so that all values are positive; β_1_). Thus, depending on the parameters β_0_ and β_1_, this function produces a probability of dropping out that is higher for participants experiencing worsening or high continued levels of symptoms and lower for participants who are experiencing benefit.

### Analysis of Simulated Data

Each simulated trial data set was analyzed using a MMRM (mixed effect model with repeated measures) to assess the significance of the biomarker-vs.-drug exposure interaction. Consistent with the recommendations of Barr et al. (28), models were initially run with maximal random effects structure justified by the design, which was then limited based on empirical success with model fits. An unstructured variance/covariance matrix was assumed.

For trial designs that include timepoints both on and off active drug excluding baseline, fixed effects in the model were time, drug-exposure, baseline biomarker and an interaction term between drug-exposure and baseline biomarker. Individual subject was included with a random intercept. The inclusion of expectancy as a fixed effect was found to increase the frequency of collinearity leading to poor model fits while changing the results minimally, and thus was not included in any of the results presented. Thus, the model implemented for these designs was: *S*_*i,t*_ = β_*i*,0_ + β_1_ · *bm* + β_2_ · *Db* + β_3_ · *t* + β_4_ · *bm* · *Db*. A non-zero coefficient for the interaction term, β_4_, serves as indication of a significant effect of baseline biomarker on drug response.

For trial designs where, excluding baseline, each participant only experiences a single treatment condition, the above model was poorly fit, and produced a significantly inflated type I error rate (data not shown). Instead, consistent with the *post hoc* analysis of a parallel group RCT's results that served as the preliminary data for this work (22), trial designs of this type (primarily OL) were analyzed with a model that included time, baseline biomarker and an interaction term between time and baseline biomarker: *S*_*i,t*_ = β_*i*,0_ + β_1_ · *bm* + β_2_ · *t* + β_3_ · *bm* · *t*, with a non-zero interaction term (this time represented by β_3_) again signifying a significant effect of biomarker on treatment response.

In each case, the model was fit using the *lmer* function from the R package *lme4* (29). For each simulated trial analysis, the *p*-value for the biomarker-vs.-drug interaction was evaluated for significance at the alpha = 0.05 level by examining the *p*-value corresponding to the interaction terms described above as calculated by *lmer*. The rate of significant interactions provided an estimate of the power of each design to detect the simulated interaction signal for each combination of parameters. The distribution bias in the estimate of the association between the biomarker and the response to active drug was quantified for each trial design and censoring pattern as the differences between the β from each replicate and the β when the analysis was run across all replicates with that parameter set but with no censoring.

## Results

**Figure 4:**
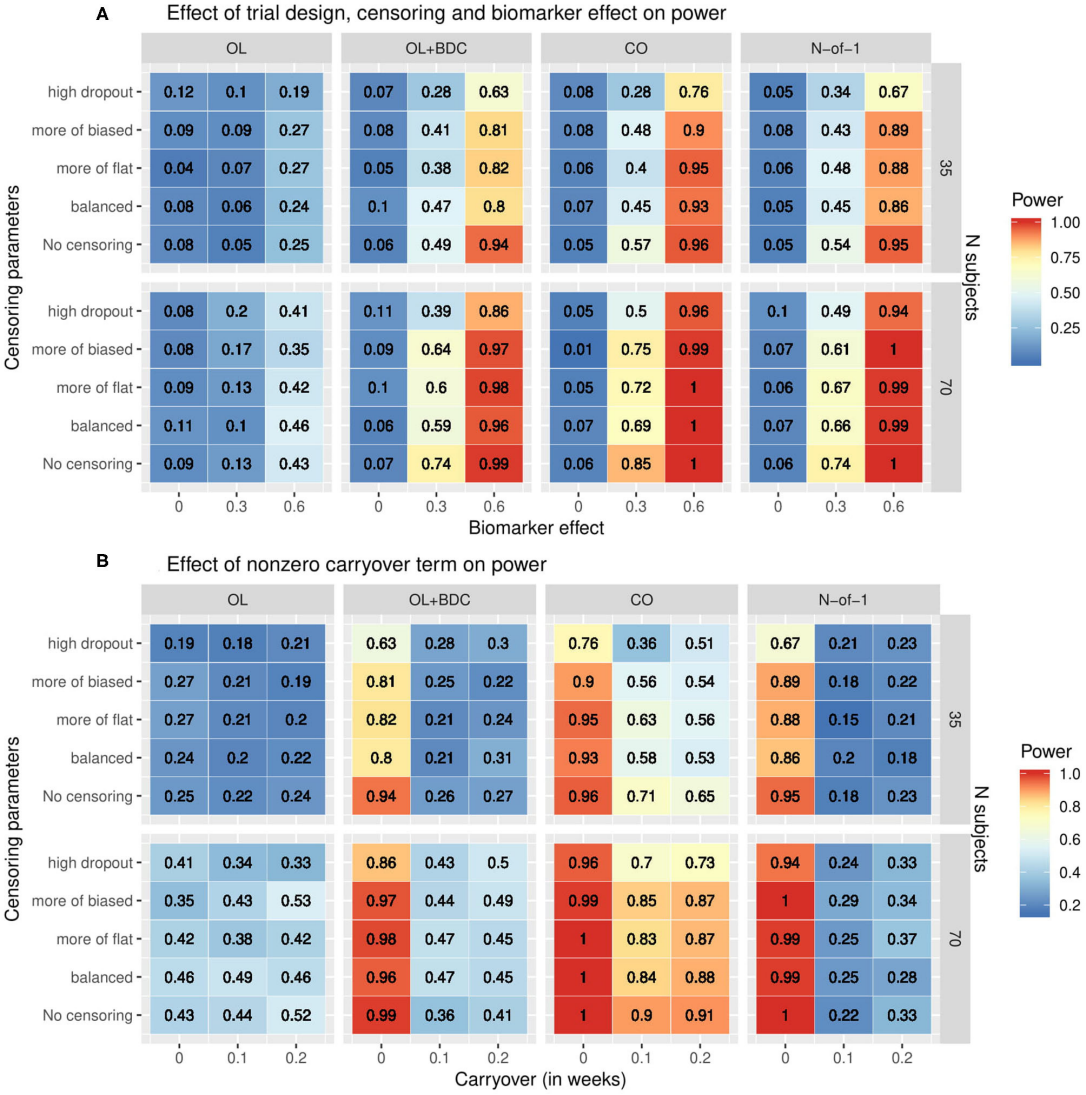
Heat map showing statistical power as a function of **(A)** clinical trial design, the number of subjects, the correlation coefficient relating the biomarker to the biologic response to drug, and the censoring parameters describing dropout patterns, or **(B)** clinical trial design, the number of subjects, the timecourse of the carryover effect of the intervention (*t*_1/2_ in weeks), and the censoring parameters describing dropout patterns, for each of the clinical trial designs described in figure 1. In **(A)** the carryover effect is set to zero; in **(B)** the correlation between baseline biomarker and the biologic response to drug is set to 0.6. OL = open label, OL+BDC = open label followed by blinded discontinuation, CO = cross over, N-of-1 = proposed N-of-1 trial design.

The statistical power to detect a relationship between the baseline biomarker and the response to prazosin treatment was significantly different among the four clinical trial designs. When simulations were run using tabula rasa parameter set, assuming equal magnitude and variance for the TR and ER factors, and without a carryover effect, the proposed N-of-1 trial design demonstrated superior power to detect a true relationship between the baseline biomarker and response to drug compared to the open label and open label + blinded discontinuation designs (Figure 4A). The N-of-1 design had lower power than the traditional crossover design. Increased censoring lowered power across all trial designs, but, consistent with the increased vulnerability of the open-label plus blinded discontinuation design to participant loss prior to the blinded component, this design's power dropped more rapidly.

### Impact of Carryover on Statistical Power

When a non-zero carryover term was added to describe the persistence of improvement related to the biologic effect of the drug even after the active drug is discontinued, described as an exponential decay with t_1/2_ measured in weeks, the presence of even a short (0.1 weeks) carryover component resulted in a precipitous decline in power in both the N-of-1 and, to a lesser but still very significant degree, the open label + blinded discontinuation design (Figure 4B). A decrease in power in the cross over design was also seen, but this was significantly less severe.

### Impact of Response Trajectory Parameters on Statistical Power

**Figure 5:**
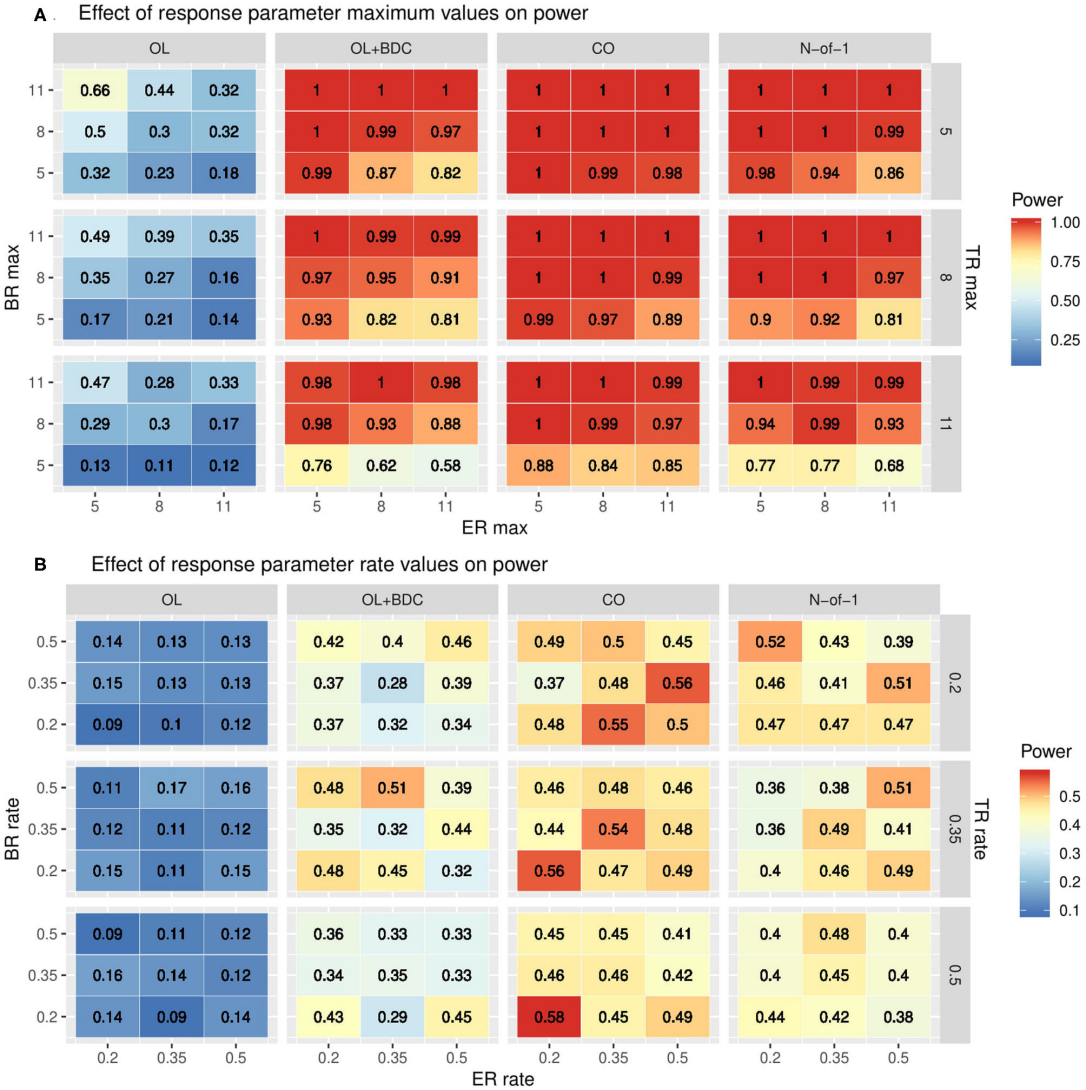
Heat maps showing statistical power as a function of trial design and the parameters used to define **(A)** the maximum response or **(B)** the rate of change in the modified gompertz function defining the trajectories of each of the three response factors (BR = biologic response to drug, ER = expectancy-related response to drug, and TR = time-varying component unrelated to drug). In panel both panels, N is set to 35 and carryover is set to zero. In **(A)** the correlation between biomarker and the BR factor is set to 0.6, while in **(B)** it is set to 0.3. OL = open label, OL+BDC = open label followed by blinded discontinuation, CO = cross over, N-of-1 = proposed N-of-1 trial design.

The impact of changes in the parameters used to define the trajectories of the three response factors (BR, TR, and ER) were explored by systematically varying either the maximum values and standard deviations (set equal to the maxima) of each factor while retaining the tabula rasa values for the rates (Figure 5A) or by systematically varying the rates while retaining the tabula rasa values for the maximums (Figure 5B). Consistent with expectation, increased maximal response of the BR factor improved power across all trial designs. Increasing the maximal response of the ER factor decreased power across all trial designs, but with a greater decrease in power in the two trial designs (OL+BDC and N-of-1) where the expectancy values changes across the trial. Increasing the maximal response of the TR factor decreased power across all trial designs. Increasing the maximal response of the ER factor decreased power more substantially for the trial design where expectancy changes.

The impact of changes in rate parameters were less consistent across trial designs. In the two trial designs with blinded discontinuation portions, an increased rate for the BR factor did generally correspond with increased power across most of the parameter space; however, for the crossover design, increased BR rate was associated with decreased power across most of the parameter space.

### Variability and Bias in Effect Size Estimates

**Figure 6:**
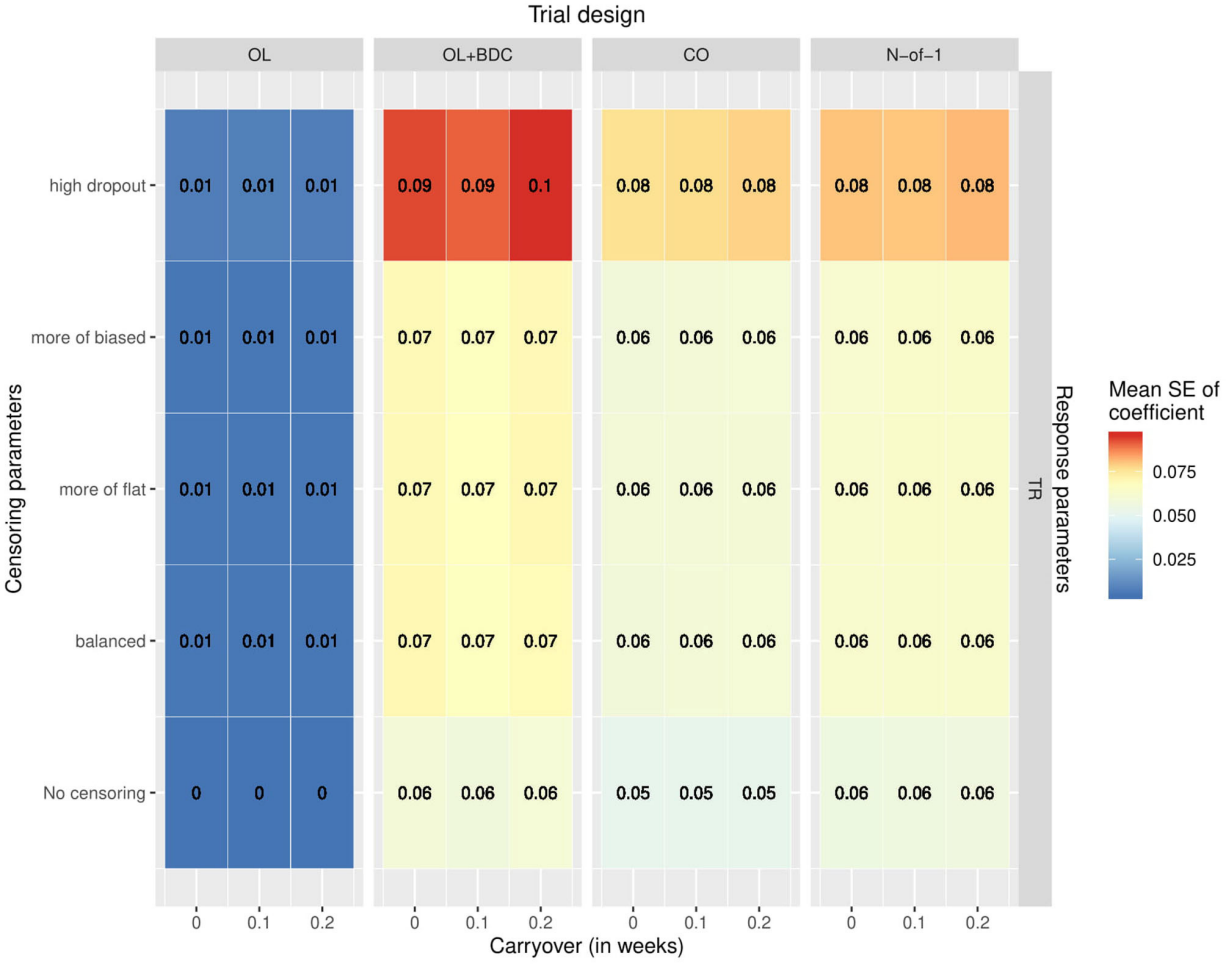
Mean standard error in the coefficient for the interaction term used for hypothesis testing across simulated replicates, as a function of trial design, response parameters, carryover, and censoring parameters. OL = open label, OL+BDC = open label followed by blinded discontinuation, CO = cross over, N-of-1 = proposed N-of-1 trial design.

The variability and bias in the effect size estimates as a function of trial design and parameters was also explored. The mean across replicates of the estimated standard error in the coefficient for the interaction term used to carry out the hypothesis testing increased across all trial designs with increasing censoring, but with a greater effect for the OL+BDC trial design (Figure 6).

**Figure 7:**
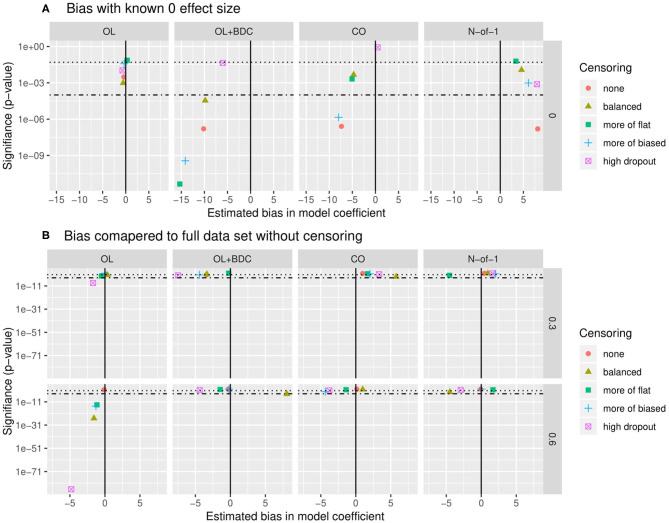
Quantification of bias in effect size estimate as a function of trial design and censoring parameters. **(A)** Bias in model coefficient for interaction term being used for hypothesis testing (β) quantified as the mean coefficient across simulated replicates when the true effect size was set to zero (estimated bias in model coefficient). *P*-value (y axis) indicates results of a two-tailed, one-sample *t*-test comparing the coefficients across the set of replicates to μ = 0. **(B)** Bias in model coefficient for interaction term being used for hypothesis testing quantified as the mean difference (Δβ) between the coefficient for a single replicate (β) and the “gold standard” coefficient for that parameter set (β_t_), with the “gold standard” defined as the coefficient calculated across all simulated participants from all replicates with no censoring. *P*-value (y-axis) indicates results of a two-tailed, one-sample *t*-test comparing the Δβ across the set of replicates to μ= 0. Dotted line indicates *p* = 0.05, dot-dash line indicates *p* = 0.0001. β and Δβ values multiplied by 1,000 for ease of visualization. OL = open label, OL+BDC = open label followed by blinded discontinuation, CO = cross over, N-of-1 = proposed N-of-1 trial design.

Bias was assessed in two ways. First, the estimate of the coefficient for the interaction term used to carry out hypothesis testing (β) was extracted for all replicates for a given trial design and parameter set but with the correlation between the biomarker and BR set to zero, and both the mean β and the *p*-value applying a one-sample two-sided *t*-test with μ = 0 to the distribution of β were examined (Figure 7A). The β-values were for most censoring patterns for the OL+BDC trial design and several censoring parameters of the CO design significantly biased toward a negative non-zero effect (*p* < 0.0001), while for the N-of-1 design, the non-censored condition showed a significant bias toward a positive non-zero effect (i.e., in the direction opposite the expected effect of a biomarker that predicts a decrease in symptoms; *p* < 0.0001).

Second, looking this time at simulations where the correlation between the biomarker and the BR response factor was set to 0.3 or 0.6, the β from each replicate was compared to the β obtained when the model was applied to all simulated participants across all replicates in the absence of censoring (Figure 7B). This analysis method allows the impact of different censoring patterns on effect size estimates to be assessed. For the open label design, for a larger true effect size, censoring was seen to result in a larger estimated effect size across all types of censoring parameters utilized (*p* < 0.0001), and in the high dropout case even with the lower true effect size. Censoring parameters did not have a significant effect on the other three trial designs.

## Discussion

These results suggest that an aggregated N-of-1 trial design beginning with an open label titration phase may provide superior power compared to an open label or open label followed by blinded discontinuation trial design, and similar but slightly decreased power compared to a traditional crossover trial design, in detecting an association between a predictive biomarker and the clinical response to the PTSD pharmacotherapeutic prazosin. In contrast to the traditional crossover design, this increased power is achieved in a clinical trial design that allows all participants to start on open-label active treatment, a significant advantage in allowing the recruitment of a symptomatic study population.

The increased statistical power seen in the N-of-1 trial design as compared with the purely open-label trial design is consistent with the information-theoretic expectation that any clinical trial design that provides minimal or no information about who in a purely active treatment group is showing a response that is specific to the intervention provided, vs. who is showing a response to treatment that is not dependent on the specific biologic treatment provided, will have an associated decrease in statistical power when used to assess the relationship of a baseline biomarker to treatment response. A significant decrease in statistical power was across all tested trial designs except the open label design when a carryover term was introduced; this effect was particularly large for the N-of-1 and open-label followed by blinded discontinuation trial designs. Although this decrease in power is consistent with expectation, the magnitude of this drop with even short half-life carryover effects underscored the critical nature of this parameter in determining the appropriateness of an N-of-1 trial design; it also suggests that the development of analysis methods that incorporate and expectation of carryover may significantly improve the power and utility of N-of-1 clinical trial designs in personalized medicine applications.

These results support both the use of aggregated N-of-1 clinical trial designs to optimize both statistical power to detect a relationship between predictive biomarkers and treatment response and clinical-logistical constraints, such as a need to allow patients to begin with active treatment. They also support the use of statistical simulation to quantitatively compare alternative clinical trial designs in such a context.

In addition to providing guidance for the design and selection of aggregated N-of-1 clinical trial designs, these types of results can help to quantify the extent to which the outcomes measured in clinical trials depend on factors such as drug carryover effects, the impact of expectancy on outcome measures, and biased dropout patterns—each of which has the potential to be highly relevant to more traditional clinical trial designs, as well.

### Implications of Carryover Effect

The impact of carryover on the design and analysis of clinical trials where participants cross from one treatment condition to another has been considered for over 50 years, and extensively researched (16, 17). In these models, we assume an exponential decay analogous to a pharmacokinetic half-life, although the simulations could be easily adapted to incorporate an alternative model. We do not assume that the half-life of the carryover effect should equal the half-life of the pharmacotherapeutic agent, however. Instead, the carry-over effect is presumed to reflect a combination of factors that includes the pharmacokinetic half-life, the time lag that may be involved in participants becoming aware of changes in their symptoms or in reporting changes on assessment tools that may have a longer lookback period, and the impact of physiologic or behavioral changes that may have resulted from changes in primary symptoms but may also serve to sustain positive changes even after the intervention has ceased. While empirical data describing the magnitude and relevance of these factors is limited for most treatments of interest, this gap in our knowledge base regarding even our relatively well-studied interventions will decrease as N-of-1 trial designs become more common. Increased characterization of the effects of discontinuing treatments has the potential to provide important clinically relevant information well-beyond its utility in the design of N-of-1 clinical trials.

### Potential for Biased Dropout

The impact of both treatment response and side effect burden on how likely different participants are to withdraw early from a trial, and at what points, is of particular importance in estimating the relative power of different N-of-1 type clinical trial designs. As is illustrated by these results, the impact on a trial of dropout rates that are biased by a participant's response to treatment has the potential to be both positive and negative. For example, the expense of running a clinical trial in which participants are enrolled for many months is significant—and if participants for whom either (a) no significant response to treatment at all, or (b) a clear response to treatment that is lost when the participant transitions back to placebo are the most likely to withdraw prior to later crossover blocks, this actually allows the additional expense of offering these extended blocks to be preferentially spent on participants for whom participation in the initial phases was inconclusive, and for whom additional blocks are the most important scientifically. This “happy accident” is of course not truly coincidental—rather, it can be seen as the result of aligning the participants' goals for trial participation (determining whether this treatment works for them, and if so, whether they need to continue to take it to maintain the effect) with the scientific goals of the trial (determining who has a specific response to the treatment that is not present with a placebo intervention).

At the same time, the potential that participants who are particularly likely to have strong placebo responses may also be particularly anxious about and likely to avoid entering discontinuation blocks is one that would decrease the power and potentially increase bias in aggregated N-of-1 trials, particularly ones that begin with an open-label titration and stabilization phase. Although the current statistical simulations do not incorporate an estimate of this type of an effect, it would be a straightforward extension do so. Additionally, as trials such as the one described here begin to be run, additional information will become available about the extent to which non-treatment-specific changes in symptoms may be associated with transitions in what the participant knows about what condition they are in (such as from open-label to blinded active treatment). This type of additional information will have the potential both to further inform N-of-1 trial design, and also help elucidate the different mechanisms and implications of non-specific treatment response. Similarly, these types of statistical models can easily incorporate the possibility of a confounding relationship between biomarkers that are putative predictors of treatment response, side effects, and actual treatment response, thus allowing researchers to assess the potential magnitude of bias in their estimates of biomarker-based predictions of treatment response as a function of drop outs biased by patterns of side effect emergences.

### Implications Regarding Placebo Response and Expectancy

One of the most complicated factors to emerge when seeking to statistically model the response patterns of participants moving between treatment conditions, and particularly between open label and blinded phases of treatment, is the expected patterns of non-treatment-specific aspects of clinical responses. By non-treatment-specific responses, we mean changes in symptoms over the course of trial enrollment that are not a result of the direct biologic action of the treatment itself—i.e., are not specific to the presence of a particular active treatment. In PG-RCTs, such effects are often grouped together under the term “placebo response,” which is used to describe all factors that together lead to changes in symptoms in the group receiving a placebo (30). This terminology is inconsistent, however, with the definition of placebo response that is used in research on the pathophysiology of the placebo effect (31), where the term is most commonly reserved for the changes in symptoms and/or physiology that are the result of a patient's expectation that they are or may be receiving an active treatment.

Here, we have considered at least four primary factors likely to contribute to the overall course of symptom change in participants: (1) the direct biologic action of the drug; (2) the average trajectory of symptoms seen as a function of time following the point at which a participant is recruited to participate in a trial (generally a regression to the mean effect, for a trial seeking to recruit acutely symptomatic, treatment-seeking participants); (3) the change in symptoms related to general factors involved in clinical trial participation, including regular contact with warm, supportive staff and ongoing monitoring of symptoms and behavioral patterns such as substance use; and (4) the change in symptoms related to the participant's belief that they are taking a medication that is likely to help them. In a typical PG-RCT, factors 2–4 are generally grouped together as the “placebo response,” and presumed to be present in both groups, with the additional impact of the direct biologic action of the medication presumed to be additive, such that it can be obtained by look at the difference between the response in the placebo group and the active treatment group (30). In the N-of-1 trial design discussed here, however, the expected timecourse of factor 4 can no longer be presumed to be static over the course of the trial, and must be modeled separately. Although this introduces additional complexity into the interpretation of the clinical trials data, it also introduces interesting additional potential analyses.

One potential benefit may be the ability to better understand the relationship of traditional PG-RCT results to the treatment effects observed in routine clinical care or in open-label trials (13). Most concretely, it has been observed that open-label contexts may result in more positive outcomes than blinded treatment conditions (32). In addition to factors such as patient selection or contact frequency, one contributing factor could be that the placebo effect is lessoned in the case of blinded treatment condition vs. open-label treatment. Additional experience with how patients' clinical outcomes differ across blinded and open-label treatment conditions may thus improve our ability to understand the relationship between the results of PG-RCTs and our clinical care contexts.

Importantly, although it is often assumed that such an effect would be linear and separable from other aspects of treatment effects, it is increasingly accepted this assumption is frequently in error, particularly for central nervous system (CNS) clinical trials (30). For example, the observation effect size and the frequency of positive clinical trial outcomes have trended downward over time as the magnitude of placebo effect in these trials has increased is frequently interpreted in the field as being due to a large placebo effect “masking” or interfering with the possibility of measuring a statistically significant treatment-specific effect (31, 33). In other words, it is being attributed to a presumed non-linearity in how treatment-specific and non-specific treatment responses combine, specifically a subadditivity—which is, in fact, consistent with emerging work on the additivity of treatment-specific and non-specific effects in clinical trials (31, 34).

There is also evidence to support the presence of interaction effects beyond subadditivity, as well. For example, in studies of two different analgesic medications operating via two different mechanisms, the treatment-specific effect was found to be either dependent on (35) or bi-directionally modulated by (36) the presence of an expected result of the intervention. In fact, such interactive effects between biologic response, non-treatment-specific effects, and even augmenting treatments are often explicitly hoped for and pursued in the context of routine clinical care (37), where a psychiatrist may e.g., remind a patient with PTSD whose treatment goals include increased behavioral activation and acclimating to attendance at anxiety-producing events that one of the expected mechanisms of action of a treatment is to allow increased ability to tolerate and learn from being present at such events. In this case, the clinician is hoping that not only does increased exposure to these activities have the potential to improve the patient's outcome both by itself and in combination with the pharmacologic treatment, but that the patient's knowledge that he is taking a medication that he expects to increase his ability to tolerate and benefit from this experience will increase his willingness to engage in the recommended activity. Although such interactive effects may significantly complicate the design and interpretation of N-of-1 clinical trials, additional experience throughout our field exploring and understanding how such factors affect patient outcomes holds the potential to make our research results more relevant to and effective for the optimization of actual clinical care practices.

The potential for complex interactive effects may also come into play in new ways as we increase the role of precision medicine methods in research and clinical care. In tests of biomarker guided treatment selection or decision making, it will be necessary to keep in mind the possibility that biomarker results may be associated not just with treatment-specific outcomes, but also with placebo response or the interaction between placebo and treatment-specific responses. For example, genetic variations in the Catechol-O-methyltransferase (COMT) gene, a key enzyme in catecholamine catabolism, has been found to be associated with the magnitude of placebo response in a variety of treatment trials (38–40). For a clinical trial such as is being modeled here, where the primary disease state (PTSD) has itself been suggested to be associated with COMT function (41, 42) and the primary hypothesis being tested is whether biomarkers of catecholamine signaling at baseline are predictive of treatment response, this suggests that the potential for interactive effects between biologic variation in placebo response, disease state, and relevant biomarkers may not be simply theoretical. Increasing use of study designs that allow increased independent assessment of expectancy-related and other non-treatment specific components of symptom change may thus become increasingly important as we seek to move toward personalized medicine models of care.

### Potential for Biased Enrollment, and Early Experience With Currently Enrolling Clinical Trial

One concern that is sometimes raised in this context is whether patients with highly distressing symptoms will be willing to enroll in a trial that involves discontinuing a what may have already been demonstrated to be an effective treatment, explicitly to see if symptoms return. Although the impact of such an effect is expected to vary significantly based on the specifics of each trial, in our experience the likelihood of this concern affecting trial enrollment is often significantly overestimated. First, those without clinical experience may underestimate the frequency with which patients in routine clinical care discontinue effective treatments to see if they still need them, with or without the awareness of their treating physician(s) (43, 44). Particularly when a treatment may require indefinite use, patients are often very interested in finding out whether any improvement they may have experienced when starting the intervention truly requires its continuation. In contexts such as antidepressant or pain management trials, where the placebo effect can be substantial, this is often a very rational question for patients to ask.

It is also possible to actively shape the likelihood that participants concerned about this possibility will avoid enrollment or not by shaping the way expectations for the duration of trial participation are conveyed. For example, in the currently running trial, ensuring a full representation of the spectrum of patients with PTSD who present for clinical care was of high priority. Thus, when the trial was described to patients, it was emphasized not only that participation was at all times voluntary, but that it was understood that at all times, the participant would need to do what was best for their own well-being—and that at times, this might mean discontinuing participation, if it turned out that symptoms exhibited substantial return during periods of discontinuation. It was emphasized that even if the potential participant were not sure if they would be able to participate in the entire trial, we would appreciate their participation for as long as it worked for them to participate. As was incorporated mathematically into the statistical modeling, it was expected that those choosing to terminate participation prior to completion of the full trial would more commonly be those for whom response to prazosin was either clearly significant or clearly minimal—while those who elected to continue for the entire trial would more commonly be those for whom it remained unclear to both participant and researchers alike whether the participant had had a significant, specific positive response to treatment or not.

Currently, the authors (RCH and MAR) are just over 1 year in to recruitment for the clinical trial (NCT03539614) that was designed based on the statistical simulation work presented here. Consistent with the concern discussed above that acutely symptomatic patients would be less likely to enroll in a trial of a widely available treatment if there were the potential that they would be initially randomized to a placebo group, and the finding that statistical power is only minimally worse for the N-of-1 design beginning with an open label period as compared with a traditional crossover design, the proposed N-of-1 design from these models was selected as the basis of the currently running clinical trial. The understanding that the trial would begin with active treatment for all participants, but that later treatment blocks would include both blinded drug and placebo, was clearly conveyed to all participants as part of informed consent. In addition to the types of outcome measures described in this simulation study, participants also completed daily symptom logs for a subset of weeks during the trial; these symptom logs included both items that were common to all participants, and items designed by the participants to reflect issues of particular importance to them in understanding how the treatment did or did not benefit them. The participants were informed at the beginning of the trial that they would be provided the data describing how their symptom reports changed during treatment with active drug and placebo at the end of the trial, and that one of our goals in the trial was to provide them as well as us as much information as possible about the ways in which the treatment did vs. did not help them, and whether they need to continue to take the medication in order to maintain any benefit that was achieved.

We found that recruitment for this type of a trial design was unexpectedly rapid, and in fact outpaced the resources we had allocated for the trial; we were eventually awarded a significant supplemental budget increase to accommodate the larger than expected recruitment interest. This experience is in contrast to multiple other PTSD treatment trials that have been run by our research group and others at our research site. The two factors that have been cited by participants and by those referring to the trial have been (1) the fact that everyone starts on active treatment, and (2) the fact that the trial is designed to provide participants with personalized information regarding their own individual response to treatment, including to what extent their symptoms were found to return when they transitioned from active treatment to placebo.

### Relevance to Clinical Trial Analysis Methods and the Development of Predictive Models of Response

One of the primary goals of clinical research into predictive biomarkers is to allow biomarker guided treatment selection. For example, if the current running trial, described above, is found to support a significant association between noradrenergic biomarkers measured at baseline and response to treatment with prazosin, the next step in testing the clinical relevance of this finding would be a clinical trial where all participants are treated with one of two active treatments, but that randomizes participants to a biomarker-guided treatment selection group vs. a non-biomarker-guided treatment selection group, and compares outcomes across the group where biomarkers are used and the group where biomarkers are not used in treatment selection.

To accomplish this, one needs to use the results of the current clinical trial to inform the development a treatment selection algorithm, which can in turn be used to guide treatment selection for individual patients. Although the focus of work presented here was on the use of statistical simulations to guide clinical trial design, the methods implemented can also be applied directly to the results of an actual clinical trial. Because the measurement of treatment response used here is continuous rather than binary, the results do not by default take the form of a classifier of individuals predicted to be treatment responsive vs. treatment non-responsive; instead, they produce a predictive model of expected mean change in total symptom severity, over a given window of time on treatment, for someone with a given combination of baseline symptom severity and biomarker measurements (an example of how to implement this analysis using existing clinical trial data is provided in vignette three in the R package accompanying this publication). This predicted response curve can then be combined with information about the expected chance of benefit and degree of benefit from an alternative treatment, along with information regarding the relative risk, cost, and convenience of both treatment options, in order to create a treatment selection algorithm for a biomarker-guided decision making trial, or for use in clinical care.

### Limitations

This work has a number of important limitations. First, the statistical simulations of clinical trials necessarily makes several simplifying assumptions, such as the presumption of linearity in combining treatment effects, the adherence of carryover effects to an exponential decay curve, the constriction of the direct, biologic response, the expectancy-related response, and non-treatment dependent effects each to a single time course, and many others. The addition of further complexity to the models has the potential for both risk and benefit. Here, where the primary goal of our statistical simulations was to guide in the selection of and power calculations for as specific predictive-biomarker clinical trial, our goal in statistical design choices was to have known oversimplifications in model implementation be at least unbiased with respect to impact on clinical trial design, and for the impact to be small or comparable relative to the degree of oversimplification in traditional power analyses. In other contexts, however, the relative cost vs. benefit of adding in or leaving out explicit modeling of different factors may be quite different.

There are also potential benefits to aggregated N-of-1 clinical trial designs that are not directly addressed in this particular set of models. For example, based on our experiences with previous clinical trial enrollment patterns, we expect there to be a significant likelihood of differential enrollment of higher acuity patients and those with a higher likelihood of being treatment responders between trial designs that begin with an open label phase and those that are entirely placebo-controlled. Although this type of differential enrollment would directly affect the power for our primary outcome, it was not explicitly included in the model.

It is also our experience from the first year of enrolling participants in this clinical trial that the opportunity to receive one's own data addressing the extent to which one (a) responded to a particular intervention, and (b) needs to remain on that intervention to maintain any observed benefit is perceived as a significant benefit by many participants, and has helped to increase not only participant recruitment but also participant engagement throughout the trial. For example, participants in the current trial have completed both medication logs and daily symptom logs at higher rates than has been observed by the authors in similar studies using PG-RCT designs (unpublished observations). This experience is consistent with previously reported assessments of patient experiences in n-of-1 trial designs (45). Such an effect might well-influence such factors as dropout and adherence, which could in turn be explicitly included in the model so as to capture their potential effect on statistical power and effect size estimation. In addition, however, these factors appear to reflect the perception by patients that participation in this type of a clinical trial design simply provides them increased personal benefit compared with participation in a traditional PG-RCT—a factors that may not directly affect power or bias calculations, but which we believe to be meaningful in and relevant to clinical trial design in and of its own right.

## Data Availability Statement

The datasets presented in this study can be found in online repositories. The names of the repository/repositories and accession number(s) can be found below: The R code and parameters extracted from pilot data that were used for this study, along with the analysis code used to generate the results presented, can be found as a GitHub package at https://github.com/rchendrickson/pmsimstats.

## Disclosure

The views expressed are those of the authors and do not reflect the official policy of the Department of Veterans Affairs or the U.S. Government.

## Author Contributions

RH and MR contributed to the conception of the study. RH, NS, and MR contributed to the initial design of the study. RH implemented the statistical simulations. RT validated this implementation. RH wrote the first draft of the manuscript. RT wrote sections of the manuscript. All authors contributed to manuscript revision, read and approved the submitted version.

## Conflict of Interest

MR is a paid advisory board member of Pfizer Laboratories, Merck, and Takeda Pharmaceuticals. RH is principle investigator of a VA CSR&D funded N-of-1 clinical trial using the methods discussed in this publication. NS has received funding from Dell Inc. These funding sources were not involved in the study design, implementation, analysis, interpretation of data, the writing of this article or the decision to submit it for publication. The remaining author declares that the research was conducted in the absence of any commercial or financial relationships that could be construed as a potential conflict of interest.
